# The neural origin of muscle synergies

**DOI:** 10.3389/fncom.2013.00051

**Published:** 2013-04-29

**Authors:** Emilio Bizzi, Vincent C. K. Cheung

**Affiliations:** Department of Brain and Cognitive Sciences, McGovern Institute for Brain Research, Massachusetts Institute of TechnologyCambridge, MA, USA

**Keywords:** motor primitive, spinal interneurons, motor modules, non-negative matrix factorization, motor cortex

## Abstract

Muscle synergies are neural coordinative structures that function to alleviate the computational burden associated with the control of movement and posture. In this commentary, we address two critical questions: the explicit encoding of muscle synergies in the nervous system, and how muscle synergies simplify movement production. We argue that shared and task-specific muscle synergies are neurophysiological entities whose combination, orchestrated by the motor cortical areas and the afferent systems, facilitates motor control and motor learning.

When the central nervous system (CNS) generates voluntary movement, many muscles, each comprising thousands of motor units, are simultaneously activated and coordinated. Computationally, this is a daunting task, and investigators since Bernstein (1967) have strived to understand whether and how the CNS’s burden is reduced to a much smaller set of variables. In the last few years we and our collaborators have searched for physiological evidence of simplifying strategies by exploring whether the motor system makes use of low-level discrete elements, or motor modules, to construct a large set of movement. In this brief communication, we argue that there is convincing evidence that the discrete elements for such simplification are muscle synergies, neurophysiological entities whose combination is orchestrated by the motor cortical areas and the afferent systems.

## EXPLICIT ENCODING OF MUSCLE SYNERGIES IN THE NERVOUS SYSTEM

The core argument for the neural origin of motor modules rests on studies of the spinal cord in several vertebral species, conducted using a variety of techniques such as microstimulation (Bizzi et al., 1991; Giszter et al., 1993), *N*-methyl-D-aspartate (NMDA) iontophoresis (Saltiel et al., 2001, 2005), and cutaneous stimulation (Tresch et al., 1999). With these approaches, we and others were able to provide the experimental basis for a modular organization of the spinal cord circuitry in the frog (with the studies cited above), rat (Tresch and Bizzi, 1999), and cat (Lemay and Grill, 2004). A *spinal module* is a functional unit of spinal interneurons that generates a specific motor output by imposing a specific pattern of muscle activation. The output of an activated module can be characterized as a force field, or the collection of isometric muscle forces generated at the limb’s endpoint over different locations of the workspace. In the spinal frog and rat, different groups of force field were activated as the stimulating electrode was moved to different loci of the lumbar spinal cord in the rostro-caudal and medio-lateral directions. Following the initial description of the force field, Mussa-Ivaldi and others found that co-stimulation of two spinal sites led to vector summation of the forces generated at each site separately. When the pattern of forces resulting from co-stimulation were compared with those computed by linear summation of the two individual fields, the co-stimulation fields and the summation fields were found to be equivalent in 83% of the cases (Mussa-Ivaldi et al., 1994). Similar results were also obtained by Hogan and colleagues (Lemay et al., 2001), Tresch and Bizzi (1999), and Kargo and Giszter (2000). Vector summation of force fields led to the hypothesis that generation of movement and posture may be based on the combination of a few discrete motor primitives. A subsequent simulation study showed that the combinations of frog hind-limb muscles that produced stable force fields were similar to the muscle groups observed to be co-activated in spinal microstimulation studies (Loeb et al., 2000). These results together argue that the experimentally derived force fields are generated by discrete groups of muscles activated as individual units, or *muscle synergies*, from whose linear combination a vast repertoire of movement and posture could be generated.

Most voluntary movements are the result of the simultaneous activation of a few muscle synergies via descending or afferent pathways which produces a complex electromyographic (EMG) pattern in the limb’s muscles. To retrieve the structures of muscle synergies from the variability of muscle activations, we and others have utilized a computational procedure, the non-negative matrix factorization algorithm (NMF), originally proposed by Lee and Seung (1999, 2001). The synergies identified by NMF are time invariant non-negative vectors whose linear combination is found, through an iterative update rule, to minimize the error of EMG reconstruction, with the additional assumption that this error follows a Gaussian distribution (Cheung and Tresch, 2005). The extracted synergies thus reflect spatially fixed regularities (Kargo and Giszter, 2008; Safavynia and Ting, 2012) embedded within diverse muscle patterns. In addition to NMF, there exist other linear factorization algorithms, such as independent component analysis (Bell and Sejnowski, 1995) and independent factor analysis (Attias, 1999). For all of these algorithms, because each synergy can have components across any subset of muscles, any one muscle may belong to multiple synergies; this aspect makes the extracted synergies different from other formulations in which each muscle belongs to a single synergy. As Tresch et al. (2006) have shown, most of the algorithms, with the exception of principal component analysis (Jolliffe, 2002), perform comparably in simulated and experimental data sets. This observation suggests the extracted muscle synergies are likely not an artifact contingent upon the particular assumptions employed by the algorithm for separating the activations of the synergies, but reflect basic aspects of muscle activations.

Recent electrophysiological experiments in the lower vertebrates, cat, and monkey have provided evidence that the temporal activations of muscle synergies identified by computational algorithms are expressions of neural activities. In the frog, discharging neurons in the intermediate zone of the spinal cord were found to be significantly related to activations of muscle synergies rather than activities of individual muscles (Hart and Giszter, 2010). In the cat, activities of distinct groups of neurons in the forelimb motor cortex recorded during reaching coincided with activations of muscle synergies identified by a cluster analysis (Yakovenko et al., 2011). In the monkey, intra-cortical microstimulation of an area of the motor cortex with descending connections to the spinal interneurons evoked EMG patterns decomposable into muscle synergies that, remarkably, matched the ones observed during natural reach-and-grasp behaviors (Overduin et al., 2012). This finding directly support the idea that the expression of voluntary movement relies on a complex pathway connecting the motor cortex and the spinal interneurons; through this circuitry, the cortex selects and combines the appropriate spinal interneuronal modules, and supplies the modules with temporal patterns of activation appropriate for the behavior being executed.

There is emerging evidence suggesting that the above conclusions may also underlie production of voluntary movement in humans. In a group of mildly to-moderately impaired stroke survivors with lesions in the motor cortical areas, we observed that the muscle synergies extracted from the stroke-affected arm were similar to those of the unaffected arm despite marked differences in motor performance between the arms (Cheung et al., 2009b). This observation is compatible with the proposal that muscle synergies are structured in the brain stem or spinal cord, and after a stroke, altered descending commands from the supraspinal areas generate abnormal motor behavior through faulty activation of the spinal modules. Similar results for the lower limb have also been presented (Clark et al., 2010). It is of course entirely possible that in humans who have developed highly skilled movements, like pianists (Gentner et al., 2010) or professional athletes (Frère and Hug, 2012), the motor cortex may also encode muscle synergies (Gentner and Classen, 2006; Rathelot and Strick, 2006). How the CNS manages to involve both the cortex and the spinal interneurons from the earliest stage of movement preparation (Fetz et al., 2002), and to integrate sensory information that contains crucial postural information related to the initial limb position during motor planning, are questions that deserve to be systematically explored.

## DO MUSCLE SYNERGIES HAVE A NON-NEURAL ORIGIN?

Kutch and Valero-Cuevas (2012) have recently proposed that the muscle synergies extracted from EMGs using factorization algorithms could have a non-neural origin. Through cadaveric experiments and computational models, these authors showed that constraints arising from the selected task and/or limb biomechanics could produce apparent couplings among muscles even when each muscle in the model is assumed to be independently controlled. This point of view that emphasizes non-neural constraints represents an important contribution to the ongoing debate on the provenance of the previously observed low-dimensionality of muscle activations (Tresch and Jarc, 2009), and offers important complementary insights. There are, however, a number of developmental and clinical studies that place the view of Kutch and Valero-Cuevas in a different light.

For instance, in a recent developmental study on human locomotor primitives, Lacquaniti and his colleagues (Dominici et al., 2011; Lacquaniti et al., 2012) demonstrated that the development of motor patterns from the neonatal to the toddler stages is primarily a result of the addition of new patterns to the few basic patterns present at birth. The precise temporal activations of all primitives are shaped gradually, over many years, as the individual grows from being a toddler to a preschooler, and finally to an adult. This progressive addition and fine-tuning of motor primitives could reflect how an infant, on his or her way to bipedal locomotion, “learns” new muscle synergies, presumably through mechanisms of associative learning and/or supervised learning, or one analogous to the mechanism responsible for the formation of ocular dominance columns in the developing visual cortex (Hubel and Wiesel, 1959). While the precise roles of genetic control in motor development remains to be established, it is conceivable that sensory feedback from muscles and tendons triggers adaptive changes in the spinal interneuronal circuitry to tune or create modules specifically tailored to the limb biomechanics of the individual, and informs other areas of the CNS of these modifications. At the termination of these developmental processes, the biomechanical properties of the limb are fully incorporated into the architecture of the motor modules, thus resulting in a match between the plant and its neural controllers that allows high-caliber motor performance. Since the limb biomechanics of different individuals are at least slightly different, it is not surprising that the precise structures of some muscle synergies are subject-specific (Torres-Oviedo and Ting, 2010). Thus, our argument that muscle synergies could have a neural origin is not incompatible with the idea that the precise structuring of each muscle synergy incorporates knowledge of both the musculo-skeletal dynamics (Berniker et al., 2009) and other biomechanical properties of the limb.

Ideally, a strong case supporting the neural origin of the muscle synergies extracted from the EMGs should come from a comparison between the number of experimentally derived synergies and the dimensionality of the space of all muscle patterns suitable for the selected tasks. In a model of the human leg comprising 14 muscles, Kutch and Valero-Cuevas (2012) (their Figure 6C) showed that the set of all EMG patterns compatible with an isometric production of endpoint forces at 16 different directions, over three force magnitudes, defined an approximately seven-dimensional subspace (with 80% of the data variance explained). On the other hand, the number of leg muscle synergies for human locomotion, including running and walking at different speeds, was estimated to be between four and five (Ivanenko et al., 2004; Cappellini et al., 2006; Dominici et al., 2011). We do not know whether, for the human leg, isometric production of endpoint forces and locomotion define spaces of motor patterns with similar dimensionalities. But the fact that the dimensionality of the observed EMGs (4–5) was lower than that expected from the constraint of a related task (7) is not inconsistent with the notion that structures such as muscle synergies of neural origin exist to constrain possibilities of motor output (Andrea d’Avella, personal communication).

There is the additional possibility that muscle synergies extracted from EMG data sets reflect regularities in the reflex and other feedback-related activities of multiple muscles arising from fixed patterns of musculo-tendon length changes that are dictated by how the muscles are arranged around the joints (Kutch and Valero-Cuevas, 2012; their Figure 1B). This is certainly an important biomechanical constraint that can give rise to apparent muscle couplings. However, in the frog, the structures of both the spinal force fields (Loeb et al., 1993) and locomotor muscle synergies (Cheung et al., 2005) persisted even after complete hind-limb deafferentation.

Another related set of findings has come from studies of muscle synergies in human stroke patients. Briefly, using the NMF algorithm and a non-negative least-squares technique, we characterized post-stroke alterations of muscle synergies in the stroke-affected arm as reflecting either a merging or a fractionation of the unaffected-arm muscle synergies (Cheung et al., 2012). Remarkably, while the extent of synergy merging correlated with the severity of motor impairment (which reflects the extent of motor cortical damage), the degree of synergy fractionation varied with the temporal distance from stroke onset (which reflects how long the motor system had been influenced by post-stroke plasticity). Given that these two patterns of synergy change correlated with variables related to the state of the nervous system, and that the biomechanical structures of the stroke-affected and unaffected arms are expected to be similar, it is likely that neural constraint is a major contributor to the structures of the observed muscle synergies in the affected arm. Alterations and merging of both upper- and lower-limb muscle synergies in stroke survivors have similarly been reported in several other recent studies (Clark et al., 2010; Gizzi et al., 2011; Roh et al., 2013).

## DO MUSCLE SYNERGIES SIMPLIFY MOVEMENT PRODUCTION BY DECREASING THE NUMBER OF DEGREES OF FREEDOM?

Muscle synergies may be conceived as representing elementary building blocks whose superposition allows the expression of a vast number of movements and postures. Similar concepts have been advanced by a number of laboratories with a variety of species ranging from *Aplysia* (Jing et al., 2004), to the frog (d’Avella et al., 2003; Hart and Giszter, 2004), rat (Tresch and Bizzi, 1999), cat (Ting and Macpherson, 2005; Ethier et al., 2006; Krouchev et al., 2006), monkey (Overduin et al., 2008), and humans (Krishnamoorthy et al., 2003; Torres-Oviedo and Ting, 2007; d’Avella et al., 2008; Monaco et al., 2010; Muceli et al., 2010). Taken together, these results indicate that *for each single task*, a reduction of the number of degrees of freedom relative to the number of muscles is a way to simplify the control of movement. In the frog, we and others have studied the activation patterns of all major hind-limb muscles collected during diverse natural motor behaviors, including jumping, in- and out-of-phase swimming, walking, kicking, and wiping. As shown by d’Avella and Bizzi (2005), each motor behavior results from a combination of both synergies shared between behaviors, and synergies specific to each or a few behaviors. While we do not know the maximum number of in-born and learned motor tasks each species may produce, it is conceivable that in any individual, the numbers of *all* task-specific and shared synergies combined may exceed the number of relevant muscles, in which case the EMGs recorded over all possible behaviors are not expected to exhibit a low dimensionality. This theoretical possibility raises the question of how muscle synergies of neural origin “simplify” movement control. We think muscle synergies simplify the production of posture and movement in the following senses. First, for tasks that can be executed by many possible trajectories or muscle activation patterns, a set of pre-existing muscle synergies can serve as a preferred channel through which the motor commands are specified. Muscle synergies thus effectively remove any musculoskeletal redundancy at the levels of posture (Santello et al., 1998; Weiss and Flanders, 2004; Bicchi et al., 2011), kinematics (Flash and Hochner, 2005), and muscle activation, by constraining how the muscles can be activated (Bernstein, 1967; Full and Koditschek, 1999; McKay and Ting, 2008).

Second, *for a single given task*, the total number of shared and task-specific muscle synergies needed for its execution is still expected to be smaller than the total number of muscles. The set of synergies thus reduces the volume of the space of possible motor commands that the CNS needs to search through by defining a subspace of a lower dimensionality. This is equivalent to a previous suggestion that preformed neural coordinative structures, such as muscle synergies, function to automatically eliminate muscle patterns that lead to uncoordinated or inappropriate movements (Tuller et al., 1982; Turvey et al., 1982). Such a reduction in search-space volume allows efficient transformation between task-level variables and muscle activations (Ting et al., 2012). This advantage conferred by a synergy-based control scheme may be particularly important for a task for which only a very small set of motor patterns is compatible with fulfilling the task requirements. For such a task, given the very large volume of the high-dimensional muscle-activation space defined by the many muscles of the limb, without any neural coordinative structures in place it would be very difficult for the motor system to discover, every time, a very small subspace of suitable motor patterns starting from any initial point in the space. The muscle synergies required could be a mixture of shared and task-specific muscle synergies either acquired through motor learning, or inherited over the course of evolution of the species (Giszter et al., 2007). Generating motor outputs by activating these synergies ensures an efficient and robust execution of a difficult task.

Third, it has been shown that the activation of each muscle synergy can accomplish a certain kinematic (d’Avella et al., 2003) or biomechanical (Ting and Macpherson, 2005) goal which may or may not be shared between behaviors. The set of all muscle synergies may then be viewed as a compendium of coordinative patterns for executing different functions that the motor system can exploit either when executing a learned task under a different dynamic environment, or when learning a new task. In broader terms, muscle synergies may be essential components in the architecture of the motor system that allow generalization to occur (Poggio and Bizzi, 2004). Consistent with this interpretation, muscle synergies were observed to be robust across very different biomechanical or behavioral contexts (Cheung et al., 2009a; Torres-Oviedo and Ting, 2010; Chvatal et al., 2011). Also, human subjects were able to adapt much faster to a perturbation if the compensatory motor patterns required could be generated simply by tuning the activations of the existing muscle synergies (d’Avella and Pai, 2010). It remains to be seen to what extent difficult and unusual movements are also executed by recruiting the synergies utilized in well-practiced behaviors.

## CAN MOTOR OUTPUTS BE GENERATED ONLY BY COMBINING MUSCLE SYNERGIES?

We have reviewed above experimental evidence that support the neural origin of muscle synergies, and argued how the combination of both shared and task-specific synergies could facilitate motor control and motor learning. We do not claim that neurally based muscle synergies are the only structures that can give rise to muscle couplings observed in experiments: feedback-related activities arising from limb biomechanics, for example, could lead to an observed coupling (Kutch and Valero-Cuevas, 2012). Nor do we claim that motor outputs can be generated only by combining a handful of spatially fixed muscle synergies. Monosynaptic stretch reflex, for instance, clearly contributes to the activities of each individual muscle. Also, at least for humans, with sufficient training even individual motor units of a single muscle could be voluntarily controlled (Basmajian, 1963). These and other additional mechanisms of motor-output generation further augment the flexibility of the motor system, and could conceivably play a role during the acquisition of motor skills (Kargo and Nitz, 2003).

One fruitful direction of future research is to determine precisely how the CNS integrates non-synergy-based mechanisms with the existing muscle synergies for the execution of a wide range of movements. In the higher primates and humans, there are two subdivisions of the primary motor cortex: a rostral, phylogenetically older region that contains descending efferents destined to the spinal interneurons, and a caudal, phylogenetically newer region that contains cortico-motoneuronal (CM) cells with monosynaptic innervations to the motoneurons of individual shoulder, elbow, and finger muscles (Rathelot and Strick, 2009). It is plausible that while the “old” motor cortex contributes to motor output by providing activation drives for the spinal modules, the “new” motor cortex further sculpts the activations of specific muscles by bypassing the spinal mechanisms through the CM cells. Controlling movement by combining muscle synergies and other proposals based on independently controlled muscles (Kutch et al., 2008; Valero-Cuevas et al., 2009) are not necessarily mutually exclusive.

## Conflict of Interest Statement

The authors declare that the research was conducted in the absence of any commercial or financial relationships that could be construed as a potential conflict of interest.
